# Which one? A suggested approach for evaluating digital health maturity models

**DOI:** 10.3389/fdgth.2022.1045685

**Published:** 2022-11-24

**Authors:** Leanna Woods, Rebekah Eden, Rhona Duncan, Zack Kodiyattu, Sophie Macklin, Clair Sullivan

**Affiliations:** ^1^ Centre for Health Services Research, The University of Queensland, Herston, QLD, Australia; ^2^ Queensland Digital Health Centre, The University of Queensland, Herston, QLD, Australia; ^3^ School of Information Systems, Queensland University of Technology, Brisbane, QLD, Australia; ^4^ Digital Metro North, Metro North Hospital and Health Service, Herston, QLD, Australia

**Keywords:** digital maturity, maturity model, digital capability, capability model, digital transformation, evaluation, digital health

## Abstract

**Background:**

Digital health maturity models allow healthcare organizations to evaluate digital health capability and to develop roadmaps for improving patient care through technology. There are many models available commercially for healthcare providers to use to assess their digital health maturity. Currently, there are limited evidence-based methods to assess the quality, utility, and efficacy of maturity models to select the most appropriate model for the given context.

**Objective:**

To develop a framework to assess digital maturity models and facilitate recommendations for digital maturity model selection.

**Methods:**

A systematic, consultative, and iterative process was used. Literature analyses and a stakeholder needs analysis (*n* = 23) was conducted to develop content and design considerations. These considerations were incorporated into the initial version of the framework developed by researchers in a design workshop. External stakeholder review (*n* = 20) and improvements strengthened and finalized the framework.

**Results:**

The criteria of the framework include assessment of healthcare context, feasibility, integrity, completeness and actionability. Users can compare model performance in order to select the most appropriate model for their context.

**Conclusion:**

The framework provides healthcare stakeholders with a consistent and objective methodology to compare digital health maturity models, informing approaches to choosing a suitable model. This is a critical step as healthcare evolves towards a digital health system focused on improving the quality of care, reducing costs and improving the provider and consumer experience.

## 1. Introduction

Digital health provides unprecedented opportunities to transform healthcare (1). Like all healthcare interventions, digital health technologies need to be rigorously evaluated to ensure they achieve improved health and care (2). Digital maturity is the extent to which digital systems are leveraged for high quality healthcare (3). Digital health maturity models are structured evaluations which allow healthcare organizations to document current digital state and develop roadmaps for improving patient care, health outcomes and health equity (4). A series of “dimensions” are often used to understand aspects of digital health capability such as business processes, organizational characteristics, information and people (5). Health service leaders can use models to track the evolution of the digital transformation process, motivate or coordinate transformation activities (6) and improve health service efficiency, effectiveness, performance and productivity (5).

National strategies outline the need to support health services in measuring and improving their level of digital maturity (7). An increasing number of maturity models are available to healthcare providers; however, it is unclear how to choose the correct model for the correct context. Importantly, existing maturity models often fail to focus on outcomes of value, but rather focus on the depth and successful implementation of the technology alone, regardless of system outcomes (2, 6, 8, 9). The importance of a maturity model that can be effectively applied to local context is emerging (6).

There is an unmet need to determine the scope and characteristics of available models (5). Our research question is: How can healthcare providers evaluate the quality and utility of digital health maturity models? The objective of this work was to create a framework to enable critical evaluation and selection of digital health maturity models by healthcare providers.

## 2. Methods

### 2.1. Setting

This work was commissioned by a federal government body, the Australian Digital Health Agency. Australia has emerging digital maturity with significant investments underway to implement digital solutions across the healthcare system.

### 2.2. Research design

**Figure 1:**
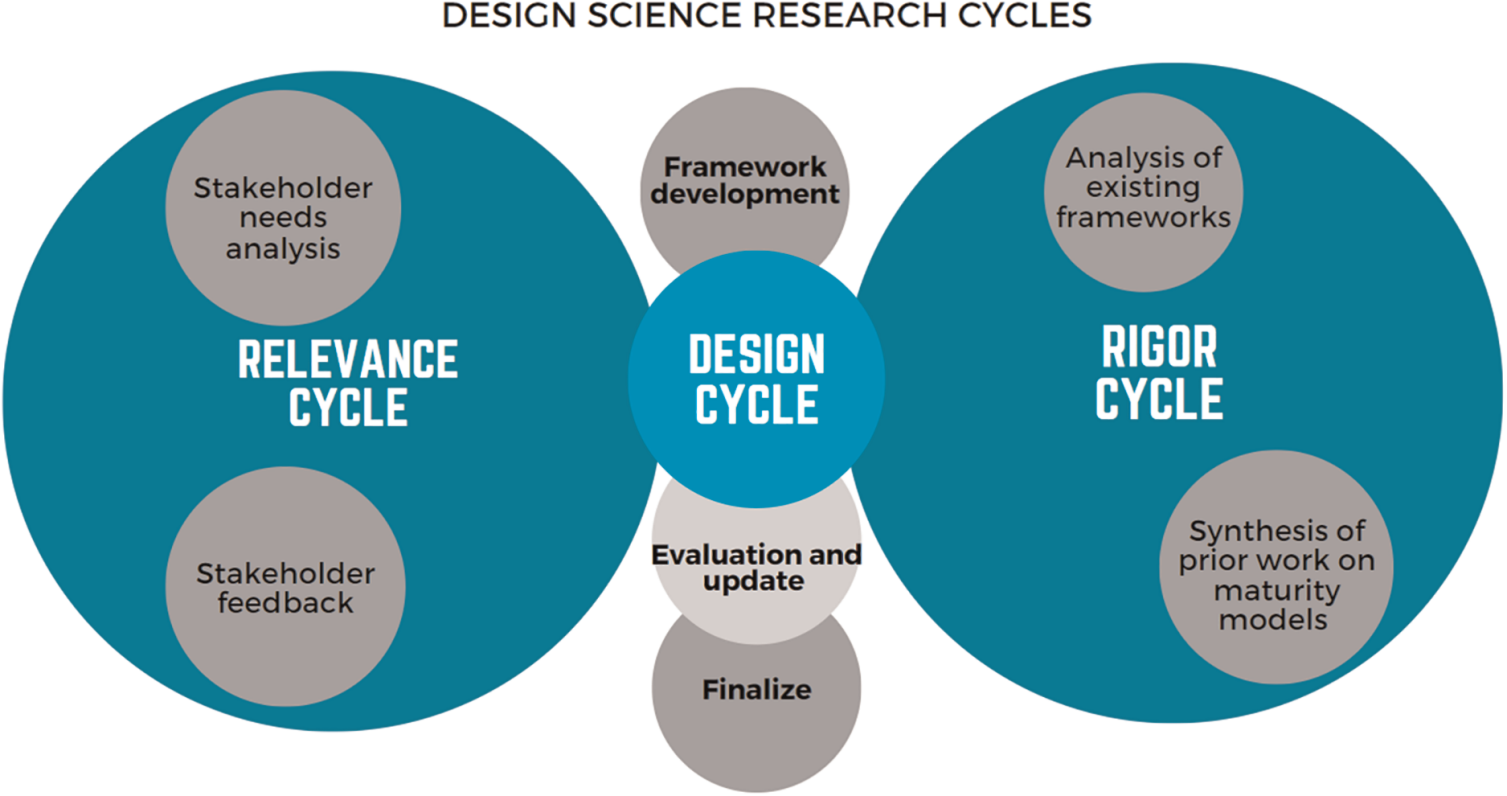
Methodological approach to the development of the framework, modified from Hevner (10).

Design Sciences Research Cycles (10) were followed to ensure the framework was developed using foundational design principles (11), consistent with the evidence-base and relevant to intended users. Three closely related cycles of activities were undertaken (Figure 1):

• The *relevance cycle* relates to the environment, comprising activities with relevant stakeholders and organizational systems or structures.
• The *rigor cycle* relates to the knowledge base, comprising activities which acknowledge the contribution of the existing literature on digital health maturity and evaluation frameworks.
• The central *design cycle* is where the framework was developed, evaluated, updated and finalized.

Activities were conducted across the following development process:

1. Content development—preliminary research activities to define the content and design considerations
2. Framework development—how the initial design of the framework was confirmed
3. Evaluation and update—review and improvement cycles to develop the final framework

Ethical approval for this research was provided by The University of Queensland [project number 2021/HE001314] prior to commencement. Participant consent was received prior to data collection.

### 2.3. Content development

Three separate research activities were undertaken to define important elements to be incorporated into the design of the framework (Table 1). Key elements for the framework development were identified through analyses in each research activity, then collated and sub-categorized into content considerations (principles to influence the content) and design considerations (principles to use for visual representation of the framework).

**Table 1:** Research activities to develop content and design for the framework.

Research activity	Research approach	Participants	Output	Design cycle
Synthesis of prior work on maturity models	Content analysis of existing digital health maturity models; 21 models identified in a government white paper (12) and 27 models identified *via* systematic literature review (9)	Research team	Content considerations	Rigor cycle
Stakeholder needs analysis	Semi-structured interviews conducted with a purposive sample from a national Interoperability Steering Committee and other digital health leaders identified through Committee membership (government, non-government organizations, academia, and industry) to understand current state and desired future state for digital health maturity models	Digital health stakeholders identified through a national Interoperability Steering Committee and purposive sampling	Content considerations and design considerations	Relevance cycle
Analysis of existing frameworks	Analysis of academic and grey literature to understand key design principles of assessment or evaluation frameworks, conducted through a modified “lightning demos” (13) research activity: identify, analyse, present, discuss and decide on key components to incorporate	Research team	Design considerations	Rigor cycle

### 2.4. Framework development

Content to be included in the framework was collated from the outputs of the research activities. Researchers translated each content consideration into a question that could be answered, and a scoring system was selected and applied. Questions were categorized into different sections for clarity and ease-of-use.

A design workshop with the research team addressed inaccuracies and gaps in the content. Design considerations informed the co-design of a visual representation of the framework. Following the workshop, the framework was updated accordingly and prepared for external consultation.

### 2.5. Evaluation and update

The framework was reviewed and improved in an iterative manner. Stakeholders who participated in the needs analysis were invited to review and submit feedback on the framework. The purpose of this review was to address any inaccuracies in content or improvements in the design and understand its perceived utility. Stakeholders submitted feedback either through a semi-structured interview with two researchers or written feedback *via* email. Stakeholders were asked to reflect and answer the following questions: What is good about the framework?; What can be improved?; Is there anything that is missing?; How do you see it being used?; What other ideas do you have?; and Is the structure correct?

Individual feedback was collated in a data table and thematically analysed by two researchers to uncover themes. Updates were incorporated into the final framework when a clear trend appeared, or when researchers agreed.

## 3. Results

### 3.1. Content development

#### 3.1.1. Synthesis of prior work on maturity models

Elements of 21 existing digital health maturity models from the grey and academic literature reported in the government white paper (12) were extracted across three categories:

• Dimensions of digital maturity
  ○ leadership and governance
  ○ workforce capability: digital literacy, clinical skills
  ○ compliance with data exchange standards
  ○ technical: infrastructure, architecture, security
  ○ patient or consumer participation
  ○ interoperability
  ○ health sector coverage
  ○ benchmarking
• New and emerging dimensions of digital maturity which are gaining prominence in recent years
  ○ user experience
  ○ innovation
  ○ organizational capability
  ○ clinical safety
  ○ adherence to government policy on data, design, infrastructure, governance and standards
  ○ efficiency
• Contextual considerations in which the models are being applied
  ○ the importance of culture in the maturation journey
  ○ the need for validation in the local context
  ○ utility by small and large health provider organizations
  ○ creating drivers for furthering organizational digital maturity

**Figure 2:**
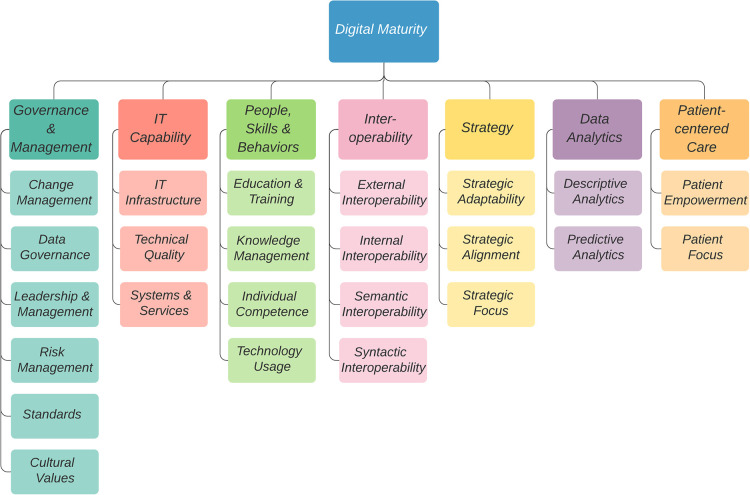
Digital maturity dimensions and corresponding indicators elicited from the literature.

Seven dimensions of digital maturity were uncovered through the systematic literature review of 27 unique maturity models (9). This was published elsewhere (9) and Figure 2 summarizes the dimensions of digital maturity and corresponding indicators used to assess dimensions:

1. Strategy: The extent to which the organization has developed and implemented a strategic plan to achieve its goals and objectives (14)
2. IT capability: The extent to which the organization has adopted and implemented IT infrastructure, digital systems, technologies, and services (15) which are usable and effective (16)
3. Interoperability: The extent to which data and information can be exchanged between systems within the organization, as well as across care settings, and with patients, caregivers, and families (17)
4. Governance and management: The extent to which the organization embraces leadership, policies and procedures, structures, risk management (quality and safety), integrated workflows, relationship building, and capacity building (18)
5. Patient-centered care: The extent to which patients, caregivers and families can actively participate in their health decisions, have access to information and health data, and co-create services and service delivery (19)
6. People, skills and behaviors: The extent to which stakeholders (internal and external) are digitally literate and motivated to leverage technology (10, 17)
7. Data analytics: The extent to which the organization uses data for effective decision making for the organization, patients, and population health (4)

#### 3.1.2. Stakeholder needs analysis

The research team interviewed 23 individuals across 16 interview sessions. Half the stakeholders were Chief Information Officers and remaining roles encompassed executives, clinicians, researchers, and state and federal government employees. Affiliations spanned government, non-government organizations, and public and private health services. The following sections report the stakeholder's and their health system's current use of maturity models, and perceptions about the ideal maturity model including design preferences, dimensions, implementation and outputs.

##### 3.1.2.1. Use and perceptions of maturity models

Most health district stakeholders reported having done some form of maturity assessment. Two models [the Healthcare Information and Management Systems Society maturity model (20) and the Victorian Digital Health Maturity Model (21)] were in use, previously used, or known by nearly half of these stakeholders. A small number of stakeholders independently reflected on their digital maturity without using a formal maturity model.

Purposes of applying existing maturity models included the need for benchmarking, driving the digital agenda, and understanding current state including gaps in maturity and priorities for change. Secondly, stakeholders emphasized providing evidence for financial decisions (return on investments or inform future investment), governance (funding, regulation, legislation), and planning for next steps (informing strategy).

Few described how maturity assessments were resourced, describing support from state government, university partners or internal resourcing. Using existing models had the benefit of minimizing procurement costs and minimized challenges associated with implementing a new process. Lack of funding was the most common reason stakeholders had not applied a model, followed by a lack of time and effort, and re-prioritization due to the COVID-19 pandemic.

Primarily, the intended purpose for applying a maturity model in the future was to inform investment decisions or apply for targeted funding. The second most common purpose was to understand the digital maturity status on the transformation journey and inform (or continue to inform) organizational strategy.

Design preferences of maturity models elicited from interview participants are summarized in descending order of importance: Simple but useful; Evidence-based; Relevant; Patient-centered; Demonstrate business value; Sustainable.

Six dimensions of digital maturity were considered by participants as most important to measure in a model, reported in descending order of importance by stakeholders: Interoperability; Level of digitalization; Data management; Infrastructure; Workforce capability; and Governance.

##### 3.1.2.2. Implementation considerations of maturity models

Most stakeholders agreed maturity models should be applied to multiple health service levels, most importantly at the jurisdiction and regional/networked levels. Stakeholders suggested different healthcare providers should use models developed for those specific settings. Overwhelmingly, stakeholders believed governments should have overall responsibility for models, and healthcare organizations themselves should conduct the digital maturity assessment.

While half the stakeholders desired an annual maturity assessment to monitor improvements, the other half reflected that the most logical frequency is every 2–3 years due to the time it takes to enact organizational change.

##### 3.1.2.3. Desirable outputs of maturity models

Consistently stakeholders desired a highly visual summary of digital maturity that was easily interpreted and facilitated the ability to compare. Stakeholders wanted the report to outline strengths, weaknesses, and opportunities based on best practice as evidence to support their improvements or impact domains. Ideally, the output would include a guideline indicating what the organization can act on to advance digital maturity.

#### 3.1.3. Analysis of existing frameworks

Key components of sampled frameworks to be considered in the design of the framework were elicited through conducting the lightning demo activity. Elements of frameworks (10, 22–29) that had potential to be incorporated included:

• Structure
  ○ reach, effectiveness, adoption, implementation, maintenance (RE-AIM)
  ○ inputs, activities, outputs
  ○ appropriateness, effectiveness, sustainability
  ○ descriptive, prescriptive, comparative
• Criteria
  ○ explanation of scores
  ○ questions asked
  ○ indicator statement
  ○ maturity levels for domains
  ○ table form with scale
  ○ criteria related to process, development, outcomes
• Context
  ○ culture
  ○ target group
  ○ health service type
  ○ impact area focus
  ○ elements of value
• Output
  ○ scores (weighted)
  ○ tally of achievement based on questions
  ○ short-term and long-term outcomes
• Design
  ○ visual
  ○ identify strength and weaknesses
  ○ spider diagram
  ○ pie diagram
• Validation
  ○ different settings
  ○ internal/external validation

#### 3.1.4. Content and design considerations

The key findings from research activities culminated in a list of content and design considerations which was used as input to the framework (Table 2).

**Table 2:** Content and design considerations which informed the framework.

Category	Element
Dimensions of digital maturity	People, skills and behaviors including education and training; knowledge management; individual competence; technology usage; user experience; workforce capability; digital literacy
	Patient centred care including patient empowerment; patient focus; patient/customer participation
	Governance and management including change management; data governance; leadership and management; risk management; standards; cultural values; innovation; compliance; adherence to government policy on data, design, infrastructure, governance and standards
	Strategy including strategic adaptability; strategic alignment; strategic focus
	Interoperability including external interoperability; internal interoperability; semantic interoperability; syntactic interoperability
	Data analytics including descriptive analytics; predictive analytics; data management
	IT capability including IT infrastructure; technical quality; systems and services; level of digitalisation; architecture; security; organizational capability
	Reflections on dimensions such as patient centred care dimension underrepresented; clinical safety and efficiency considered outcomes not dimensions; dimensions of maturity in hospitals only (what about primary care?); interdependencies between dimensions remains unclear
Content considerations	Digital maturity assessment process possibly hybrid model of self-assessment and independent body
	Recommendations are actionable, easily interpreted, useful
	Acknowledgement of the maturation journey in the organization (e.g., help inform investment decisions and organizational strategy, apply for funding, guideline to advance maturity, demonstrate business value, frequency aligned with digital strategy, recognize foundations to progress, understand current state)
	Highlight priority areas including strengths, weaknesses, opportunities
	Credible, evidence-based with evidence to support impact domains
	Transparent method of assessment as the model may be proprietary
	Sustainable, repeatable, easy to resource including self-assessment capability
	Acknowledgement of the maturation journey outside the organization (e.g., external benchmarking, national benefit, ability to compare with peer organizations)
Design considerations	Criteria including explanation of scores or tally of achievements based on questions
	Relevant to the healthcare context, applied to multiple health service types, large/small organizations and validated
	Simple in structure, possibly highly visual with specific structures suggested

### 3.2. Framework development

A design workshop and internal review of Table 2 (summarizing content and design considerations) facilitated the development of the initial version of the framework. The document was prepared for external consultation.

### 3.3. Evaluation and update

Twenty stakeholders provided feedback. Stakeholders reported that the version was well-structured, responding positively to the individual sections and the length. Stakeholders reported the framework was comprehensive yet generalizable to varied health services, simple and easy-to-use. Most responded positively to both the presence of the scoring system itself and the 0, 1, 2 nature of the scoring system. However, most stakeholders were uncertain as to how to interpret the final score, namely because the total achievable scores varied across the different sections.

Several comments suggested incorporation of new and emerging concepts in healthcare and digital health technology (e.g., prescriptive analytics, wearables, artificial intelligence, virtual care). Multiple individuals commented that the focus should be patient and population outcomes. Feedback included broad suggestions to update wording or include additional content. Wording and content were updated when a clear trend emerged in the data, or when all researchers agreed on the update. Broadly, stakeholders also suggested the following general principles when updating the framework document:

• Ensure any instructions presented are clear and concise
• Update any technical language to be more easily understood
• Consolidate phrases as needed. Separate questions as needed
• Update the language to be more generic, such that it is not hospital focused
• Update the language to be more active and aspirational
• Update the language to be more patient and population focused

These principles were incorporated, and a user guide was added for additional clarity.

### 3.4. Framework to evaluate digital health maturity models

The purpose of the framework is to provide healthcare stakeholders a consistent and objective methodology to compare maturity models identified by different vendors.

**Figure 3:**
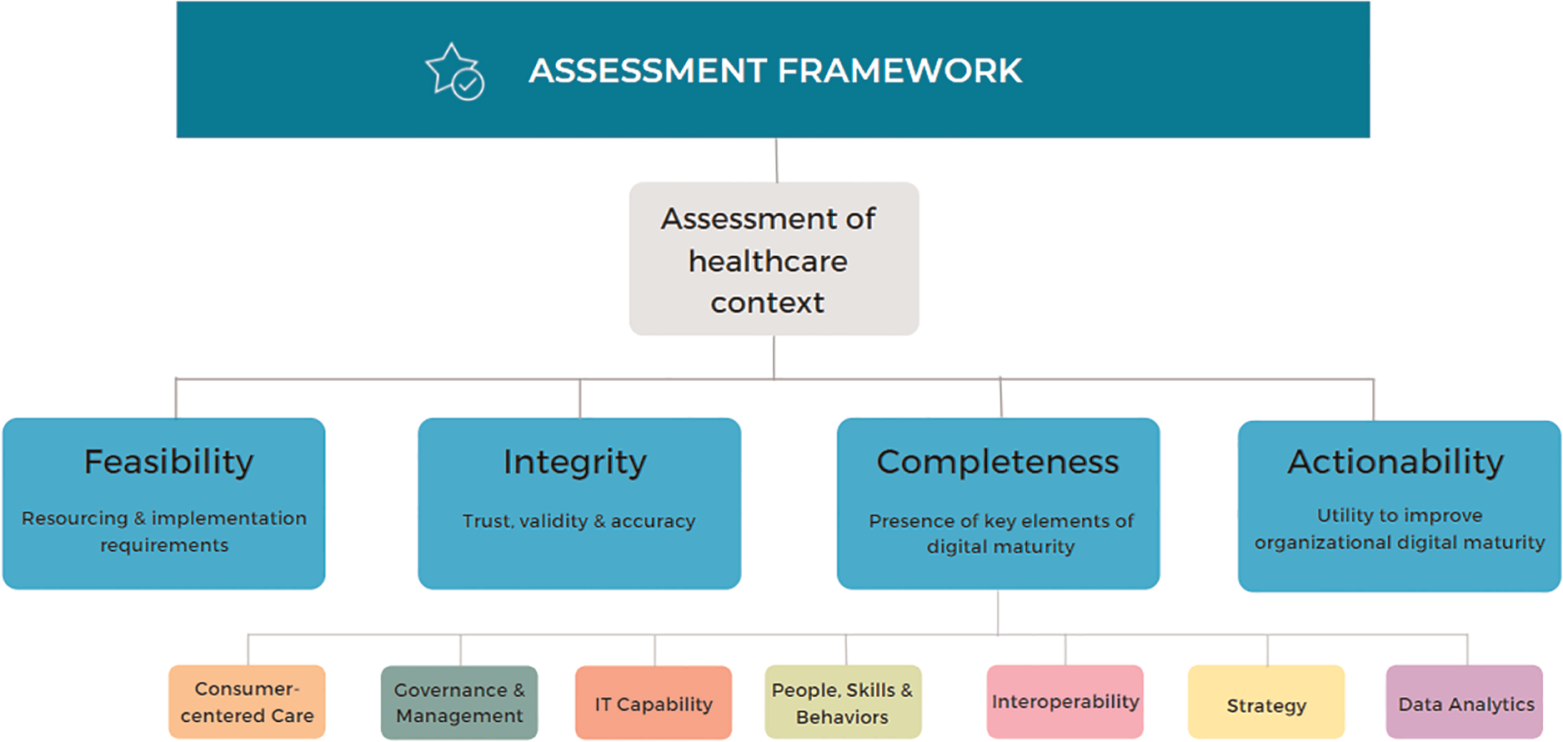
Framework to evaluate digital health maturity models.

The framework contains five sections (see Figure 3 and 'Supplementary Data Sheet 1 for the full framework):

1. Assessment of healthcare context: to understand to which healthcare contexts the model could be applied. If the model cannot be applied to the appropriate healthcare context, the user may decide against proceeding with the assessment.
2. Feasibility assessment: to understand the model’s resourcing requirements and the organization’s ability to secure those requirements. It also considers the implementation requirements, accessibility of collected data, and vendor commitments to improving the model over time.
3. Integrity assessment: to evaluate the extent to which the results can be trusted and considered as accurate assessments of an organization’s digital maturity.
4. Completeness assessment: to understand the extent to which the model considers critical elements of digital maturity. Seven dimensions and 24 indicators of digital maturity were identified by a systematic literature review, which was refined to seven dimensions and 27 indicators through stakeholder consultation. This section assesses the presence and extent to which the model addresses the dimensions and their respective indicators.
5. Actionability assessment: to understand if the results from the model can be used to improve healthcare outcomes, and capacity for internal and external benchmarking.

Remaining sections contain criteria in the format of a question. Questions can be answered using the following scale: 0 = No; 1 = Somewhat/Maybe; 2 = Yes; U = Unknown. Calculating subtotals across the sections enables identification of the strengths and weaknesses of the model. After completing the assessment for several maturity models, users can compare model performances to provide recommendations for maturity model use.

## 4. Discussion

Healthcare providers need assistance with their digital transformation strategies. We heard clearly from healthcare stakeholders that they require the results from applying a model to identify gaps in digital health, identify future directions for growth and help inform the business case for digital health investments. The framework to evaluate digital health maturity models can help healthcare providers select an appropriate model based on feasibility, integrity, completeness and actionability.

Applications of digital health maturity models are scarcely described (30). The process of applying the framework prompts users to seek responses to criteria, facilitating a transparent and fair evaluation of quality. As consulting firms and non-government organizations market maturity models, a method for healthcare providers to choose an appropriate model is important. Vendors will increasingly need to be transparent with the content of their models to enable evaluation.

The correlation between digital maturity and healthcare outcomes is limited (3) and needs investigation. Current focus on technology implementation depth, rather than health outcomes need to evolve. Further work is needed to correlate technology implementation and quality improvement measures such as healthcare outcomes mapped to the quadruple aims of healthcare—better patient experience, better clinician experience, improved health of the population and reduced cost of care (8).

A major benefit of utilizing this framework is to guide and enable the implementation of appropriate maturity models. In doing so, health services on all levels have the ability to benchmark, both internally and externally, improving their overall digital maturity and facilitating comparison against their peers. It is still unknown if a single maturity model can be applied to multiple healthcare contexts, so further work is needed to work towards recommending maturity models for use. A recent review uncovered that the most common scale that models are applied to is “multiple hospitals” (5). Applying the framework to multiple models will uncover the model(s) most relevant to the targeted context. This remains an important next step.

### 4.1. Limitations

Consistent with the consultative design methodology, stakeholders involved in the design and development of the framework were purposively sampled and deliberatively influenced its content. The results are therefore potentially biased and not necessarily generalizable to different healthcare settings (13). Systematic review methods to identify and evaluate existing digital health maturity models reported in the academic literature were required to complement the government white paper. We performed several steps to improve credibility of the results, including transparency of data collection and analysis, stakeholder review with 20 individuals and acceptance by the commissioning government agency. Additional review by additional healthcare providers would be helpful to validate the framework.

### 4.2. Conclusion

Digital transformation is now an essential component of the strategies for all healthcare organizations. A digital health maturity assessment can assist healthcare providers with their digital health strategy and monitor progress towards achieving organizational goals through digital change. Currently, it is unclear how to choose a digital health maturity model. We have developed an evidence-based framework to enable assessment and comparison of digital health maturity models. This is critical step as digital health systems evolve to focus on improving the quality of care, reducing costs and improving the provider and consumer experience.

## Data Availability

The original contributions presented in the study are included in the article/**Supplementary Material**, further inquiries can be directed to the corresponding author/s.
