# Supplementary material for: Which one? A suggested approach for evaluating digital health maturity models
Source: Front Digit Health. 2022 Nov 24;4:1045685. doi: 10.3389/fdgth.2022.1045685 (PMC9731136; doi:10.3389/fdgth.2022.1045685)
Supplement: Supplementary file 1 [file Datasheet1.docx]

Framework to evaluate digital health maturity models

# Assessment of healthcare context

In this section, assess to which healthcare contexts this maturity model could be applied.

| What model is being assessed? | |
| --- | --- |
|  | |
| At which scale does the model assess digital maturity? | Yes |
| National health system |  |
| State/territory health system |  |
| Regional/networked health system |  |
| Individual healthcare facility |  |
| Individual practice |  |
|  | |
| At which health service type does the model assess digital maturity? | Yes |
| Hospitals (private and public hospitals, including associated ambulatory care in outpatients and emergency departments when located in the same facility) |  |
| Primary care (e.g., general practice) |  |
| Community based care |  |
| Mental health facilities |  |
| Indigenous health services |  |
| Diagnostic/imaging/laboratory facilities |  |
| Pharmacies |  |
| Other (e.g., aged care, social care, allied health, ancillary health services) |  |

# Feasibility

In the following sections, assess the feasibility to apply the maturity model using the following scale:

0 – No

1 – Somewhat/Maybe

2 – Yes

U – Unknown

| 1 | Feasibility Criteria | Score | | | |
| --- | --- | --- | --- | --- | --- |
|  |  | 0 | 1 | 2 | U |
| 1.1 | Identify the lifetime cost for the model (e.g., initial, implementation, recurring fees). Consider costs can be negotiated. Are the costs reasonable relative to value of applying the model? |  |  |  |  |
| 1.2 | Can the organization secure the required external resources (e.g., external consultants) to implement the model? |  |  |  |  |
| 1.3 | Can the organization secure the required internal resources to implement the model? |  |  |  |  |
| 1.4 | Can the data required for the model be readily be obtained by the organization? |  |  |  |  |
| 1.5 | Is the process to apply the model clear? |  |  |  |  |
| 1.6 | Is the data collected as part of the model stored and accessed in a way that is consistent with organizational policies and procedures? |  |  |  |  |
| 1.7 | Does the vendor have a demonstrated track record of updating the model? |  |  |  |  |
| SECTION SUBTOTAL | | /14 | | | |

# Integrity

| 2 | Integrity Criteria | Score | | | |
| --- | --- | --- | --- | --- | --- |
|  |  | 0 | 1 | 2 | U |
| 2.1 | Has the model been used locally? If yes, note the healthcare context below. |  |  |  |  |
| 2.2 | Is the content relevant to local healthcare providers (e.g., reference national standards)? |  |  |  |  |
| 2.3 | Has the model been validated (e.g., peer-reviewed literature, industry groups)? |  |  |  |  |
| 2.4 | Is the model evidence based (e.g., grounded in the peer-reviewed literature, industry recognized best practice)? |  |  |  |  |
| 2.5 | Does the model tie digital maturity to tangible healthcare outcomes (e.g., quadruple aim of healthcare) which is evidenced by data? |  |  |  |  |
| SECTION SUBTOTAL | | /10 | | | |

# Completeness

The completeness section assesses the presence and extent to which the model addresses the seven key dimensions of digital maturity and their respective indicators.

In this section, determine if the model assesses the following elements of digital maturity, using the scale below:

0 - This model poorly assesses this indicator OR this model does not assess this indicator

1 – This model adequately assesses this indicator

2 – This model comprehensively assesses this indicator

U – Unknown

| 3.1 | Consumer-centered Care: The extent to which consumers, caregivers, families and communities are partners in their own care, design and develop new services and have fair, equitable access to inclusive care based on their preferences | Score | | | |
| --- | --- | --- | --- | --- | --- |
|  |  | 0 | 1 | 2 | U |
| 3.1.1 | *Consumer Enablement:* The extent to which consumers are partners in their own care, and generate, access, receive and use relevant information and health data |  |  |  |  |
| 3.1.2 | *Consumer Engagement*: The extent to which the organization partners with consumers and incorporates patient-reported outcome and experience measures to design and develop new models of care |  |  |  |  |
| 3.1.3 | *Equitable Access:* The extent to which consumers and populations have fair, equitable access to care that is inclusive, safe, based on their preferences and closer to home |  |  |  |  |

| 3.2 | Governance and Management: The extent to which the organization embraces leadership, policies, procedures, structures, risk management (quality and safety), integrated workflows, relationship building, and capacity building. | Score | | | |
| --- | --- | --- | --- | --- | --- |
|  |  | 0 | 1 | 2 | U |
| 3.2.1 | *Change Management*: Ability to encourage individuals and teams to embrace planned change to achieve desired outcomes |  |  |  |  |
| 3.2.2 | *Data Governance*: How data integrity, security, privacy, confidentiality, and accessibility are preserved across the digital systems and healthcare setting, supported by standardized processes, and authentication and authorisation protocols |  |  |  |  |
| 3.2.3 | *Leadership and Management*: The executive team’s commitment to improve clinical quality and foster innovation through leveraging digital technology |  |  |  |  |
| 3.2.4 | *Risk Manageme*nt: The need for the workforce to identify, mitigate, and report risks ensuring the safety, security, and privacy of consumers and the workforce |  |  |  |  |
| 3.2.5 | *Standards*: The extent to which processes, policies, and procedures are based on the national and international healthcare standards that have been formally agreed, mandated, and contribute to optimising the healthcare organization |  |  |  |  |
| 3.2.6 | *Cultural Values*: Encourages innovative behaviors within a trusting and inclusive environment |  |  |  |  |

| 3.3 | IT Capability: The extent to which the organization has adopted and implemented IT infrastructure, digital systems, technologies, and services which are usable and effective | Score | | | |
| --- | --- | --- | --- | --- | --- |
|  |  | 0 | 1 | 2 | U |
| 3.3.1 | *IT Infrastructure:* Infrastructure and architecture designed and implemented to support the systems and services |  |  |  |  |
| 3.3.2 | *Technical Quality*: Effectiveness, efficiency, fit for purpose and usability of digital systems |  |  |  |  |
| 3.3.3 | *Systems and Services*: Digital systems implemented to support clinical care delivery via efficient and effective processes (e.g., electronic medical records, clinical decision support systems, eprescribing, picture archiving and communication systems, orders and results management, asset and resource optimisation systems, and remote and assistive care systems) |  |  |  |  |
| 3.3.4 | *Cyber Security:* Ability to protect the system against both physical and virtual security threats and adapt to evolving security threats |  |  |  |  |

| 3.4 | People, Skills and Behavior: The extent to which stakeholders (internal and external) are digitally literate and motivated to leverage technology | Score | | | |
| --- | --- | --- | --- | --- | --- |
|  |  | 0 | 1 | 2 | U |
| 3.4.1 | *Education and Training:* Opportunities to grow and develop digital literacy through clinical, technical, information, collaboration, teamwork, leadership and decision-making skill development (e.g., role descriptions, credentialling, development plans) |  |  |  |  |
| 3.4.2 | *Knowledge Management*: The extent the workforce capability grows through creating, managing, and sharing knowledge |  |  |  |  |
| 3.4.3 | *Individual Competence*: Considers individuals need to possess skills, knowledge, and capability to use digital systems to support safe, effective care. Measures the digital literacy of executives, clinicians, and consumers |  |  |  |  |
| 3.4.4 | *Technology Usage*: Recognizes that systems can be used in different ways, and that digitally mature organizations need to ensure systems are used as intended in a pervasive and consistent manner |  |  |  |  |

| 3.5 | Interoperability: The extent to which data and information can be meaningfully exchanged between systems within the organization, as well as across care settings, and with patients, caregivers, and families | Score | | | |  |
| --- | --- | --- | --- | --- | --- | --- |
|  |  | 0 | 1 | 2 | U | |
| 3.5.1 | *External Interoperability:* Integration of systems, services, and data across the entire healthcare system including national systems and use of national healthcare identifiers, to enable exchange of meaningful information |  |  |  |  |  |
| 3.5.2 | *Internal Interoperability*: Integration of systems and data across departments within a single healthcare organization to enable exchange of meaningful information |  |  |  |  |  |
| 3.5.3 | *Semantic Interoperability*: Ability of information exchanged between digital systems to be accurately interpreted and understood by each system involved which is dependent on the transparency of the underlying lexicon and data dictionary |  |  |  |  |  |
| 3.5.4 | *Syntactic Interoperability*: Technical standards have been defined and adopted to enable the consistent, effective, and efficient integration of digital systems, services and exchange of information |  |  |  |  |  |

| 3.6 | Strategy: The extent to which the organization has developed and implemented a strategic plan to achieve its goals and objectives | Score | | | |
| --- | --- | --- | --- | --- | --- |
|  |  | 0 | 1 | 2 | U |
| 3.6.1 | *Strategic Adaptability:* The organization’s strategy and its digital systems are dynamic, capable of responding to both environmental challenges and emerging opportunities |  |  |  |  |
| 3.6.2 | *Strategic Alignment*: The digital strategy is aligned with the organization’s strategic landscape; grounded on clinical benefits and recognizes people, process, information and technology |  |  |  |  |
| 3.6.3 | *Strategic Focus*: Emphasis on quality, safety, sustainability, cost effectiveness, and ensures the systematic evaluation of quantifiable results and objectives |  |  |  |  |

| 3.7 | Data Analytics: The extent to which the organization uses data for effective decision making for the organization, individuals and populations | Score | | | |  |
| --- | --- | --- | --- | --- | --- | --- |
|  |  | 0 | 1 | 2 | U | |
| 3.7.1 | *Descriptive Analytics:* The analysis of data to identify and understand historical patterns and trends, facilitating effective decision making |  |  |  |  |  |
| 3.7.2 | *Predictive Analytics*: The analysis of data that enables future potential risks and opportunities to be identified to aid decision making, including proactive or predictive models of care (e.g., artificial intelligence) |  |  |  |  |  |
| 3.7.3 | *Prescriptive Analytics:* The analysis of data extends to extraction and communication of recommendations about clinical risk in practice through clinical decision support |  |  |  |  |  |
| SECTION SUBTOTAL | | /54 | | | |  |

# Actionability

In this section, assess the actionability of the maturity model using the following scale:

0 – No

1 – Somewhat/Maybe

2 – Yes

U – Unknown

| 4 | Actionability Criteria | Score | | | |
| --- | --- | --- | --- | --- | --- |
|  |  | 0 | 1 | 2 | U |
| 4.1 | Does the maturity report communicate results clearly? |  |  |  |  |
| 4.2 | Does the maturity report provide practical, useful recommendations to drive improvements? |  |  |  |  |
| 4.3 | Can the model be applied at a frequency that aligns with the organization’s strategic plan? |  |  |  |  |
| 4.4 | Can the model be applied at a frequency that allows for internal benchmarking to track progress over time? |  |  |  |  |
| 4.5 | Does the model provide the opportunity for comparison against peers? |  |  |  |  |
| 4.6 | Can the maturity report be customized? |  |  |  |  |
| SECTION SUBTOTAL | | /12 | | | |
| TOTAL | |  | | /90 | |

Results summary

Using this template as a guide, summarize your evaluation of the digital health maturity model. The subtotal of each section can be weighted in presenting these final results, with weights to be determined by the assessor to reflect the priorities of the organizations that will be using the maturity model.

Assessment framework

| Section | Result |
| --- | --- |
| Assessment of healthcare context |  |
| Feasibility |  |
| Integrity |  |
| Completeness |  |
| Actionability |  |
| TOTAL |  |

Critical reflection summary

Provide a brief description of the model and a narrative on each assessment criteria (healthcare context, feasibility, integrity, completeness, actionability).

|  |
| --- |

Overall impressions

Provide your impressions of the maturity model or recommendations for use.

|  |
| --- |
